# The Last Sentiments of Suicides

**Published:** 1851-07-01

**Authors:** A. Brierre de Boismont


### THE LAST SENTIMENTS OF SUICIDES.

BY DR. A. BRIERRE DE BOISMONT.

*(Translated from the Author's MSS.)*

### II. BAD SENTIMENTS.

MAN's moral station may vary in form, although the fundamental conditions remain the same. Joy and sorrow, pleasure and pain, good and evil, these are the elements of his destiny, which separately, in connexion or in combination, constitute the circle from which he cannot escape.

In a former article we reviewed the better sentiments expressed by suicides; we will now consider the darker and more painful category of bad sentiments.

This second class comprises the analysis of seven varieties of expression, comprising 615 cases (474 men and 141 women). At first sight there seems but a slight difference between this class and the preceding, but that is owing to our having considered as blameable all motives put forth to justify disgust and weariness of life; although, as we have shown elsewhere,* these manifestations of exaggerated sentimentality are closely allied to insanity. It is also worthy of remark that, even among those who destroy themselves from weariness of life, very many express good sentiments.

The class we are about to analyze may be divided into five sub-sections.

1st Sub-section.—*Expressions of discontent, of anger, of hate, &c.; complaints, reproaches, insults to their enemies, persecutors, representatives, parents; to the world at large, to persons by whom they consider themselves aggrieved. Reflections on the misery of human life. Imprecations, declamations, concerning their troubles, trials, and misfortunes.*

To suffer and complain is the common lot of humanity. But complaint may be gentle and lowly, or it may be loud and querulous, reproachful, or full of invective and menace. 304 documents (217 men, 87 women) contain variable shades of this sentiment. The most common subjects of complaint

* A. Brierre de Boismont, "Histoire Statistique Médicale et Philosophique du Suicide." (In the press.)

are of a domestic character, coming from children and parents, husband and wives, lovers and mistresses, &c. The sum of these documents may be thus divided:—Domestic motives, 51; legitimate or illegitimate alliances, 122, (marriages, 63, concubinage, 59); friendship, 2; 129 writings, whilst expressing the sentiments of the writers, and the cause to which they attribute their death, refer to no person in particular, and seem to have originated solely in the desire of making known their misfortunes.

In the male sex, complaint against their families comes chiefly from young men, frequently in reply to reproaches previously addressed to them. A boy, fourteen years old, writes on the shutter, "Adieu of — I., who hanged himself to his mother's curtain because she was always scolding him for idleness." Brothers complain of being neglected by their brothers; young persons accuse their stepmothers; others reproach their fathers for not allowing them sufficient money, or for ill-using them. Parents, in their turn, deplore the misery entailed on them by the debauchery and misconduct of their children. Young women complain of not being allowed to marry the object of their affections, of the cruelty of their parents. Mothers deplore the ill-treatment received from their husbands, and sometimes from their children. Many women, by their jealous, scolding, quarrelsome, mischievous dispositions, render the lives of their husbands quite insupportable.

A father, horribly outraged, writes a letter to his young son while still a child, with injunctions not to open it until sixteen years old. In this letter he makes a frightful and overwhelming revelation respecting the boy's mother, and concludes by giving him his curse if he does not avenge him on the authors of his woes. The Attorney-general ordered this letter to be destroyed.

Occasionally the alleged motives are utterly false; a man represents himself as the victim of the wife, whom he has betrayed and abandoned, and overwhelms her with insults. The infidelity of a mistress is a frequent cause of death. Sometimes an indifference to the objects of life leads to suicide. "You would not comprehend me," says one of these unfortunates; "if I had possessed a fortune, I should perhaps have been more happy! To struggle as far as in me lay against my destiny, I have just staked my life in an hour's play at the Palais Royal; I have lost, so I have nothing left but to die."

Husbands, by their ill-treatment, their infidelities, and shameless presentation of their concubines, fill the souls of their wives with despair. "For the last thirteen years," one of them writes, "my husband has not ceased to ill-use me, beating me frequently, infecting me with filthy disorders, and constantly, when intoxicated, threatening to kill me. Twice he has thrown me down, and knelt on me, and some happy chance has alone prevented his assassinating me. Such a life is intolerable. I have suffered too much, and now prefer death." "Infamous wretch!" exclaims another, "you and your paramours will end miserably. You have made me the most unhappy of women. I leave you ruined,"—and animated by the desire of vengeance, she burns all the linen, destroys all objects of value, breaks the furniture, throws into the fire bank-notes to the amount of £500, together with promissory-notes, and other papers; terminating her maledictions with these words: "nothing, nothing more, to quench your passions." The abandonment of their lovers, their marriage, their indifference and contempt, are fertile sources of suicide with females. With the intention of exciting the jealousy of a lover, whose tenderness seemed diminishing, his mistress informs him that she is about to get married; he answers quietly that she could not do better. Driven to despair at this, she writes: "I expected that you would feel annoyed at the prospect of my union with another, and far from encouraging the project, have dissuaded me from it; but since it is not so, I am about to arrange my affairs so as to embarrass you no longer."

The complaints of suicides relate to all kinds of subjects: illness, misfortune, mankind, creditors, humiliation, injustice, calumny, masters, employers, landlords, tenants, neighbours, &c. &c., are designated as causing the fatal deed. One individual, who had failed of success in everything, invokes death for the king and all in power, as tyrants and wretches. A musician pretends, that the conduct of the leader of the orchestra, to which he belongs, is the cause of his death. "If I had not been ten minutes behind time, he and his business would have been settled before mine, but he will be touched in a more sensitive part." One man writes—"I die, in order that my daughter may not have a murderer for her father; for, if I lived much longer, I should certainly blow out the brains of the villain who has so shamefully deceived and robbed me."

From among these numerous letters, the varying tones of the sad chords of human sorrow, we extract the following passages, purposely omitting all vague complaints, imprecations, and curses on society. "I have lived a victim; I die a philosopher. Mankind is pervert; want overtook me; it was my duty to escape from it. I wish for no priest. My linen must be sold to pay expenses. In no way do I regret what I have done. I have a profound conviction that, before a hundred years are passed away, all the earth will be a universal republic." "I have been without work for some time past—I have not eaten for two days—that is why I kill myself." "Forgive my secret sorrow, I have lost all joy and happiness; you have enjoyments, I regrets; you live, but *I have lived.*" "Not being able to overcome the misfortune which has always pursued me, life has become a burden too heavy for me. When this is found, I shall have ceased to live: Oh, pity me!"

> "Donec eris felix multos numerabis amicos;

> Tempora si fuerint nubila, solus eris."

"I have 6860 francs to pay to-day; I have only 660 francs; I want, therefore, 6200 francs; where shall I find them? how obtain them? I already owe more than 30,000 francs, so all is lost."—"A villanous usurer, who has the names of more than 300 substitutes on his list, has done me out of all my money; I have lost my freedom for no profit; I now prefer death."—"The loss of my fortune in 1830; repeated disasters since, have deprived me of all energy, and the threat of my landlord to turn me out of my lodging is the finishing stroke. I recommend my poor sick wife to the kind attention of the charitable. I wish to be buried in the churchyard, and pray that a mass may be said for the repose of my soul."—"On the one hand, two possessions of some value, a few years since, but now, in consequence of competition and new inventions, become insufficient to afford me an honourable independence (and my ambition did not exceed that;) on the other hand, the vexation of not being able to pay certain debts in full; to find myself, at fifty-four years of age, without employment and without funds, with no other means of subsisting than by descending to some servile employment, to which my character would never submit; and, worse than all, my health impaired, and growing weaker day by day. Such are the principal reasons which have driven me to self-destruction. May God forgive me."—The preceding was written in a firm, strong hand, an hour only before the fatal act was committed. —"Consumed with grief at the successive loss of all who were dear to me, I could no longer live in isolation and solitude. Looking to the future with horror, I have decided to rid myself of a life which was a burden to me. I request that I may be buried without any pomp."—Two sisters write: "If we took a lover, it was because our labour did not yield us enough to live on. We wished to bring up our child. Besides, we have done only what so many do, without having poverty for an excuse."

Unfortunately, the present system of education does little or nothing towards impressing the *sentiment of duty*, and it is owing to our having such vague and confused notions on this important point, that so great anarchy reigns in our moral and intellectual world, and that we see among us so much indifference and frivolity in the gravest matters. The illustrious Coleridge, whilst visiting the Vatican, saw two French officers approach the statue of Moses. "I'll wager," exclaimed the poet to his companion, "that their first remarks will be on the rays and beard." It was so. "What an old goat," said one; "And cuckold," said the other. "It is strange," remarked Coleridge, "that the French are the only creatures in human shape who can understand nothing of art or religion."

2nd Sub-section.—*Ennui, disgust of life; it is lawful to rid oneself of it when it becomes burdensome.*—Ennui is not a fiction. It is ofttimes the shadow of humanity. We find it given as the cause of suicide in 237 writings (192 men, 45 women). Out of this number, ennui is, in 138 cases, connected with some of the other causes enumerated, but in 99 cases is the only cause stated. Having already gone fully into this subject, we need not here repeat what we have said.

*Useless on the earth; a burden.*—The majority of persons who destroy themselves have an idea that they are useless alive; that they are merely an encumbrance and burden to others. This sentiment is particularly common among those who commit suicide from real misfortunes or long sickness. Four letters (three men, one woman,) express this idea. The following is an extract from one of them:—

"When I started in life, I was alone, without fortune, without friends, but filled with youthful ardour; I manfully engaged in the struggle, and, for a time, success crowned my efforts; but, with increasing years, and the charge of a family, misfortune and ruin came upon me. I had grown aged, and I found out what a useless encumbrance an old man is to all about him. Of no good to my family; a burden to myself; wounded in my dearest affections, nothing remained for me but to die; so I have made up my mind to do it."

This letter is clearly written, without exaggeration or aim at effect. The other three express the same sentiments.

3rd Sub-section.—*Materialism, scepticism, indifference.*

Although the sentiment of religion exists in the majority of mankind, there are some individuals who, either by a vicious education, or by some natural defect, or perversity of character, seem entirely destitute of it. Twenty-nine letters (twenty-eight men, one woman) state the absence of this grand principle. The expression varies, but the same idea of nothingness exists in all. "For some time past," writes one, "I have longed to sleep a profound sleep. After so much suffering and fatigue, I shall at last find repose."—"Having never possessed either wit or talent," says another, "I do not see any necessity for me to vegetate thirty or forty years here below; besides, what matter twenty years sooner or later, since it comes to the same thing at last; I prefer finishing the business at present. If I had any sentiment of love in my heart, I should perhaps have resisted. And, after all, what is death, since all end with our life."—This writes: "Death is a sleep which knows no waking;"—that quotes the famous couplet—

"Quand on a tout perdu, et qu'on n'a plus d'espoir,
La vie est un opprobre, et la mort un devoir."*

These lines occur in several letters.

Sometimes the transition from life to death is marked by a lively fancy. "I have just left my friends, who are going to a ball, whilst I am listening to the crackling of my ardent orchestra. What an odd contrast! it is a comedy which terminates in a doze." With certain suicides, the memory of some great pleasure is the culminating point—the "ne plus ultra" of existence. "After having enjoyed the love of my mistress, I have nothing more to do but to die. What could I feel beyond that? Is the world worth living for? I have spent eight days in debating the question. There is neither folly, nor courage, nor cowardice, in killing oneself; it is a simple matter; when life displeases, you grow tiresome and insupportable." Many affirm that the dead alone are happy, and manifest no regret for their deed; their greatest disappointment would be not to die; they invite their friends to come and inspect their remains, and learn how people kill themselves; and nothing is more common than to find them asserting that death is an eternal sleep! The only woman who affirmed death to be the remedy and oblivion of all our ills, had no moral principles whatever; she had once already been confined in a house of correction for misconduct: on the eve of her suicide and of her marriage, she had risen from her bed, and stolen forth to meet one of her lovers. Being threatened by her parents with another imprisonment, she wrote a letter to them, saying that she wished merely to live in freedom after her own fashion; that she was tired of their constant scoldings and remonstrances; that she would rather return into nothingness than be thwarted in her desires.

*Insults to the clergy.*—The Voltairian spirit, whose exaggeration has done so much mischief by extinguishing the principle of religion, without which no nation can long exist, is manifested in a considerable number of letters, by the express request of the writers, that their bodies may not be taken near the church, but at once carried to the cemetery. Sometimes the disregard for religion is carried still farther: for instance, in one letter, not only is the priesthood insulted and vilified, but religion itself is represented as the most cruel foe of humanity.

4th Sub-section.—*Thoughts of vice and debauchery.*—The depravity of morals, which must not be confounded with the perversion of instincts, is not arrested even in the face of death. Many official inquiries reveal the fact, that men sometimes commit self-destruction amidst all the refinements of sensuality. Sometimes, however, there is a real perversion of the faculties, as in the case of the madman, about twenty years ago, who bribed the girl with whom he was cohabiting to stick a knife into his neck at a stated moment; which she, being largely paid, actually performed, inflicting several wounds, and for which she was condemned to ten years' imprisonment.

We may also cite the instance of another madman who succeeded in persuading a prostitute to castrate him during the venereal orgasm. At the time we were engaged in reporting and editing the clinical lectures of the celebrated Dupuytren,† we saw in his consulting-room a man who seemed extremely feeble, and who was using his handkerchief to stop the

* In opposition to this French couplet, we recal an English one equally famous:—

"When all the blandishments of life are gone,
The coward creeps to death, the brave lives on."—Tr.

† Brierre de Boismont et Marx. "Leçons Orales de Cliniques Chirurgicales faites à l Hotel Dieu, par le Baron Dupuytren." 6 vols. in 8vo. Paris.

bleeding from a wound in his scrotum. The wound was a longitudinal incision on the right side, and, on examination, the testicle of that side could not be found. The wounded man stated that he had been caught in the act of adultery, and that the husband had inflicted that punishment on him. However, on further examination, the surgeon discovered that the other testicle also was wanting; and, on closely questioning his patient, obtained, amidst much prevarication and self-contradiction, a confession, that the mutilation had its origin in a monstrous perversion of the sexual instinct, and was not the effect of jealousy. We find in nine writings (seven men, two women) details which leave no doubt upon the depravity of mind which attended their authors in their last moments. A workman writes to some prostitutes, "What a glorious party we shall have; it shall be my last spree."

Our friend, Dr. Forget, informs us that he was called on by the police, about a year since, to verify a suicide committed under singular circumstances. A man, still young and well dressed, accompanied by a young female, entered one of the most celebrated restaurateurs in Paris, and asked for a private room. He ordered a choice dinner and the best wines, so that the bill came to forty francs. Immediately after dinner he arose from his seat, went to a corner of the room, and inclining his head slightly to one side, put a pistol to the temple, and blew out his brains. When picked up he was dead. The woman, on being questioned, stated, that she had met her companion for the first time on the preceding evening, when he had proposed the dinner at the restaurant. That when he called for her, he seemed calm and self-possessed; that he ate and drank very heartily, was gay and loving, and that there was nothing in his conduct to give her any suspicion of his fatal design. On searching him, it was found that he had nothing in his pockets.

5th Sub-section.—*False motives.*—It has been remarked that life is a long play, in which no personage appears without a mask. Whilst we object to the universal acceptation of this opinion, we are forced to admit that falsehood is the order of the day, and we are ready to repeat with the witty diplomatist—"La langue a été donnée à l'homme pour déguiser sa pensée."* In this land of vanity every one seeks to attract attention, and this pretension does not yield even to death.

If hypocrisy is the homage which vice pays to virtue, it is no wonder that so many disguise themselves in that livery. Thirty-one documents (twenty-six men, five women) afford us evidence on this point. Among the alleged motives of suicides we often find complaints against their families—"My wife, my children, my relations," writes one man, "are the authors of all my misfortunes; they have never ceased to poison my existence. I worked night and day for them, and they repaid me with disgust. By seizing on all my savings, they have reduced me to extreme poverty. Death will soon deliver me from my tormentors, to all of whom I bequeath my curse." The inquiry showed that this unfortunate wretch had always been a bad husband, a bad son, and a bad father, and that he had destroyed himself to avoid the pursuit of justice, consequent on his attempt to violate his own daughter. Another addresses to his brother, the managing director of an important administration, a letter couched in

* The parentage of this sarcastic saying has recently been disputed by a lively French writer, and assigned to the late eccentric manager of the Porte St. Martin Theatre. In reality, however, it belongs to a greater genius than either, namely, Oliver Goldsmith. "Men of the world," says he, in one of the papers of the "Bee," "maintain that the true end of speech is not so much to express our wants, as to conceal them."—"Oliver Goldsmith, a Biography," by Washington Irving, chap. 22.—Tr.

the following terms:—"You would not present me to your minister because I was badly clothed, and you too proud to acknowledge any relationship with a poor devil like me. It was easy for you to have procured me the means of living honourably, but your egotism would not admit of it. All for yourself, nothing for others, that's your motto. Yet, notwithstanding your ingratitude towards me, I wish you well, and freely forgive you my death." Now, turn over the page, and we find that this generous victim was an idle, worthless *débauché,* a gambler, always getting into debt, who, in return for numberless acts of kindness and assistance repeatedly afforded, wreaked, by this posthumous calumny, vengeance on a brother, of whose virtues and success he had always shown himself basely jealous. And, unfortunately, the malice of such a shaft may serve as a barb to make it stick, and so embitter the existence of an honest man, whose misfortune it was to have so vile a brother. A father accuses his son of causing his death by his cruelty, whereas, it was notorious that he had dissipated not only his own property, but also the son's fortune. A husband reproaches his wife with having rendered his life miserable by her shrewish disposition; whereas, it was clearly established by the testimony of all who knew them, that the wife was a very gentle, affectionate woman, the husband a confirmed drunkard.

A young man pretends to justify his act by saying that his mother had been prejudiced against him by persons who had squandered his inheritance, whereas it turned out that he had been disinherited by his mother for a gross insult, and that he had destroyed himself because he no longer possessed the means of continuing his riotous course of living.

Sometimes suicides ascribe their fatal resolution to the ill-luck which they assert has always pursued them. "I kill myself," says one of these, "without having to blame myself for a single fault which should have led to this fatal determination. Wine, women, and play have never had any attractions for me. In fact, I was always fond of work. I leave the three shillings, which is all I possess, to the poor." The writer of this epistle was a gambler, drunkard, and débauché, who had attempted to murder his wife.

There are some who seek to ascribe their death to the influence of a passion, culpable in the eyes of religion and morality, but which sometimes obtains pity for those who are its victims. One of these expresses himself in the following manner:—"I cannot overcome my attachment to a married woman, as good as she is faithful; nevertheless, an imperious necessity compels me to avoid her for ever. Why should the institution of marriage be thus perverted by our social conventions? Adieu, dear angel, my only comfort upon earth." The investigation revealed that the said angel was a common prostitute, who refused to renounce her occupation, and had for some time kept the man who thus complains of the injustice of society. The preceding instance recalls to mind the history of a notorious villain, executed some years since in Normandy, for having strangled several people, who, when on the scaffold, beside the instrument of death, and about to appear before God, solemnly exclaimed, "It is now impossible to lie—I declare I am innocent."

Ofttimes vanity induces the suicide to invent some romantic tale, with the view of creating a fictitious interest in his fate. A young man appears before a commissary of police, and states that he had been attacked by several individuals, stripped, forced to swallow poison, and afterwards cast into the water in the Champs Elysées. At first his story obtained some credit, but on inquiry it was discovered that the pretended victim was an idle, worthless fellow, of very extravagant tastes, overwhelmed in debt and embarrassments, who had doubtlessly himself committed the injuries of which he accused others. It was found that he had actually taken poison.

Some seem impelled to suicide by the desire of escaping from the bad reputation which their evil actions have procured them. A woman writes that a certain party, whom she designates for the execration of all honest persons, wishes to ruin her and her children, and has made an attack on her honour, which is dearer to her than life. It turns out that this Lucretia had shamefully abused the confidence placed in her by her employer, and was likely to fall into the hands of justice for embezzlement.

A man who destroyed himself with a garland of "*immortelles*" on his head, after declaiming against the miseries of life, requests to be interred with a medal of Faith about his neck, near to the angelic sister who will receive him in heaven. He protests that he is the victim of a conspiracy, and strongly denies a theft which had been clearly proved against him.

Some suicides attempt to give a false interpretation to the mode of their decease, pretending an accident, assassination, &c., in order to escape the ignominy attached to self-murders, and so save their families from disgrace and sorrow. A merchant writes a letter, partly gay, partly serious, very well calculated to deceive the public as to the manner of his death; but in a second letter addressed to his family, he acknowledges that he kills himself in consequence of the overthrow of his hopes and the loss of his fortune.

Résumé. The affective sentiments, wounded self-love, express themselves by recriminations and complaints, by insults and threats. These various sentiments follow an order in relation to the moral organization of man; the family occupies the first place, then come husbands and wives, lovers, mistresses, and society at large.

In the family, parents attribute their despair to the evil doings of their children, and their misconduct; children complain of the continued reproaches addressed to them; of the persecution of a step-mother; of the avarice of their parents; of their constant harshness; women complain of not being allowed to marry the men they love; of the cruelty of their parents; of ill-treatment from their husbands, sometimes from their children.

As regards marriage, the frivolous, extravagant, shrewish, jealous, quarrelsome disposition of wives, and their infidelities, frequently occasion the suicide of husbands; whilst husbands, by their immorality and debauchery, their violence of temper and ill-usage, often render the lives of their wives insupportable.

As relates to concubinage; the abandonment of lovers, their indifference, neglect, or marriage, cause the suicide of their mistresses. The same motives affect men equally.

The complaints of suicides are based on all sorts of motives—false, true, serious, trivial, futile; frequently the real cause remains concealed. In short, suicides accuse themselves, others, society in general, and the world at large.

All the writings in the second section have a closer or more distinct reference to ennui and weariness of life. This sentiment may be inherent or acquired, accidental or congenital. Accidental ennui is due to the influence of sickness, poverty, misfortune, losses, sorrow. Congenital ennui is allied to the natural character and disposition of the individual,—to his temperament, his organization,—in a word, to his humour. It commences in early life, and may continue to an advanced age. Ennui may also depend on an incurable indolence and indifference of character, which makes employment of all kind unendurable, and all pursuits tiresome. Among the ennuyés may also be placed many restless, envious, jealous, frivolous spirits, who declare implacable war to society. Many who destroy themselves from weariness of life leave beside them books containing apologies for suicide, often opened at the most striking passage. Some are merry in their last hour. Others declare that their death inflicts no wrong on any one—that their life was useless.

Disgust of life is less marked among women, owing to their greater hopefulness, their religious sentiments, their stronger affections and domestic habits.

The analysis of the third section is devoted to the irreligious sentiments. It would seem that the man who is about to destroy himself ought to have renounced all ideas of another life. This is the case in this series, which includes professions of materialism, invocations of oblivion, and, as a natural consequence, insults addressed to religion and the clergy. But here, as elsewhere, good and evil are blended, for we find many documents which attest the religious belief of the writers.

The fourth section contains facts which prove that man's evil instincts do not always quit him in the supreme hour of his fate.

Finally, the last section is devoted to the investigation of false motives. This chapter, so full of instruction, shows us that men sometimes die with a lie on their lips. We find individuals explaining and excusing their suicide by motives apparently sincere, plausible, and affecting; and on inquiry we find these pretended victims to have been villains, debauchés, thieves, bad sons, bad husbands, and bad fathers. Theirs is hypocrisy in death. Sometimes the suicide wishes to spare the feelings of his family, or avoid the opprobrium of self-destruction, and gives a false explanation or fictitious colouring to his action.

(*To be continued.*)

---

